# Auxin Signaling Mediated Spatial Accommodation Mechanisms During Lateral Root Development

**DOI:** 10.1111/ppl.70481

**Published:** 2025-09-02

**Authors:** Kevin Bellande, Cristovāo De Jesus Vieira Teixeira, Joop E. M. Vermeer

**Affiliations:** ^1^ Laboratory of Molecular and Cellular Biology, Institute of Biology University of Neuchâtel Neuchâtel Switzerland; ^2^ IPSiM, University of Montpellier, CNRS, INRAE, Institut Agro Montpellier France

**Keywords:** auxin, cell wall remodeling, lateral root development, spatial accommodation

## Abstract

Spatial accommodation, the ability of plant tissues to adapt structurally during organogenesis, is important for the successful growth and emergence of new organs, such as lateral roots, through overlying cell layers. This process requires precise coordination between cellular architecture and physical as well as biochemical signals. As a key determinant of root system architecture, auxin is also an important regulator of spatial accommodation. Of these responses, the modulation of the cytoskeleton dynamics and cell wall remodeling across multiple tissue layers is of particular importance. Here, we focus on how auxin signaling controls these dynamics to enable tissue‐scale plasticity during lateral root emergence. We also compare how these mechanisms vary across plant lineages, highlighting the differences between dicots and monocots, but also non‐vascular plants.

## 1 Spatial Accommodation in Root Systems

Spatial accommodation is a multifaceted concept operating across multiple biological scales, from organ‐level organization to precise cellular adaptations (Karlova et al. 2021). The ability to dynamically adjust spatial organization in response to organogenesis and environmental changes is essential for maintaining tissue function and resilience. Unlike animals, plants depend on continuous post‐embryonic organogenesis to adapt their growth and morphology and optimize fitness in response to fluctuating environmental conditions (Banda et al. 2019; Beckers et al. 2025; Dastidar et al. 2012; McCleery et al. 2017; Motte and Beeckman 2019). Root system architecture (RSA), which defines the spatial configuration of roots within the soil, plays an important role in this plasticity (Smith and de Smet 2012; Morris et al. 2017). The development of RSA begins with the formation of roots during embryogenesis, progressing through the initiation and growth of lateral roots (LRs), root hairs, and higher‐order root structures (*Plant Roots: The Hidden Half, Fifth Edition*—*Google Books*; Beeckman and Eshel 2024). In dicot species such as *Arabidopsis thaliana* (Arabidopsis), RSA is typically characterized by a dominant primary root from which lateral roots branch. The decision to branch or not to branch is a key determinant in modulating the RSA. In contrast, monocots develop a root system comprising primary, seminal, and crown roots, which emerge at different developmental stages (McSteen 2010; Shekhar et al. 2019). The phytohormone auxin, specifically indole‐3‐acetic acid (IAA), acts as a master regulator of these adaptive processes that coordinate root branching, cell elongation, and tissue remodeling. Here, we explore how auxin mediates spatial accommodation during LR development. We also examine how these auxin‐dependent mechanisms differ in complexity between dicots, monocots and non‐vascular plants, highlighting potential evolutionary adaptations and tissue organization.

## 2 Orchestrating Lateral Root Development: The Central Role of Auxin Signaling and Transport

As a master regulator of the RSA, auxin is required for both initiation and emergence of LRs as well as root angle orientation (Du and Scheres 2018; Roychoudhry and Kepinski 2022). The spatial distribution of auxin within roots plays a crucial role in determining cell fate and patterning of tissues (Overvoorde et al. 2010; Chen et al. 2021). The auxin signaling network evolved from ancestral regulatory modules during the early stages of terrestrial colonization and has since diversified and become more complex (Hernández‐García et al. 2024; Vanneste et al. 2025). In Arabidopsis, LR initiation is governed by a tightly regulated auxin signaling network. Intracellular auxin is perceived by auxin receptors belonging to the TRANSPORT INHIBITOR 1/AUXIN AUXIN‐RELATED F‐BOX PROTEIN (TIR1/AFB) family and co‐receptors of the transcriptional repressor protein family of AUXIN/INDOLE‐3‐ACETIC ACID (Aux/IAA) proteins. Upon binding of auxin, the Aux/IAA proteins are degraded via the proteasome, thereby derepressing the AUXIN RESPONSE FACTOR (ARF) transcription factors to modulate the expression of genes essential for LR development (Lavenus et al. 2015; Voß et al. 2015). Recent work further reveals that TIR1/AFB receptors also act as adenylate cyclases, producing cAMP as a crucial second messenger required for ARF‐mediated transcriptional reprogramming and auxin‐regulated developmental processes (Chen et al. 2025). These signaling modules are evolutionarily conserved across land plants (Bowman et al. 2021; Yu et al. 2022; Hernández‐García et al. 2024). LRs originate from founder cells in the xylem pole pericycle (XPP), and their initiation and emergence depend heavily on local auxin concentrations and signaling intensity (Cavallari et al. 2021). In addition, extracellular auxin is perceived by AUXIN BINDING PROTEIN 1 (ABP1) together with ABP1‐LIKE PROTEIN 1 and 2 (ABL1 and 2). This module is required to activate proteins belonging to the TRANSMEMBRANE KINASE (TMK) family, and these are also required for proper cell division patterns during LR development (Huang et al. 2019; Yu et al. 2023). Increased IAA levels correlate with elevated LR density, whereas mutations in auxin transporters, such as PIN‐FORMED (PIN1, PIN3, PIN4, PIN6, and PIN7) or influx carriers (AUX1 and LAX3), as well as in signaling components like ARF7 and ARF19, result in reduced LR formation (Casimiro et al. 2001; Benková et al. 2003; De Smet et al. 2007; Okushima et al. 2007; Swarup et al. 2008).

LR emergence requires the primordia to grow through multiple cell layers, the endodermis, cortex, and epidermis, without compromising the integrity of the primary root (Vermeer et al. 2014; Vilches‐Barro and Maizel 2015). Not only does auxin coordinate LR development but it also regulates the responses of overlying tissues, facilitating spatial accommodation (Stoeckle et al. 2018). Disrupting auxin signaling or alterations in the mechanical properties of surrounding cells can delay or impair LR progression (Ditengou et al. 2008; Lucas et al. 2013; Marhavý et al. 2013; Vermeer et al. 2014; Vilches Barro et al. 2019). Turgor pressure and cell wall mechanics create mechanical conflicts between the expanding LR and the surrounding cells. This requires fine‐tuned adjustments to the composition and thickness of the cell wall (Mirabet et al. 2011; Hamant and Haswell 2017; Moulia et al. 2021). Several studies have emphasized the importance of mechanical forces in modulating LR development, with Sutcliffe and Sexton (1968) being among the first to link glycerophosphatase activity in *Pisum sativum* roots to the pressure exerted by the emerging LR. Auxin facilitates this process by regulating aquaporin expression, thereby influencing water flow and turgor pressure dynamics in LRs and adjacent tissues (Péret et al. 2012; Reinhardt et al. 2016; Sager et al. 2020).

At the tissue level, auxin enables adaptive modifications in RSA by integrating hormone signaling, cell‐to‐cell communication, and spatial patterning (Banda et al. 2019). For example, in response to soil moisture gradients, roots exhibit hydropatterning, which promotes LR formation on the side of the root exposed to water, and xerobranching, which suppresses LR emergence in dry air gaps (Bao et al. 2014; Orman‐Ligeza et al. 2018; Orosa‐Puente et al. 2018; Mehra et al. 2022; Roy et al. 2025). Hydropatterning is driven by asymmetric auxin signaling, which is regulated by ARF7 SUMOylation and reactive oxygen species (ROS) (Orosa‐Puente et al. 2018; Roy et al. 2025). In contrast, xerobranching involves abscisic acid (ABA)‐mediated plasmodesmata closure, which inhibits auxin movement. Auxin and ethylene jointly modulate root responses to water availability; ethylene suppresses LR development on air‐exposed surfaces via proteins such as FASCICLIN‐LIKE ARABINOGALACTAN‐PROTEIN 4 (FLA4), which may perceive mechanical or environmental signals (Scharwies et al. 2025).

LR spacing is determined by local auxin maxima in founder cells, which are formed in the elongation zone (EZ) of the PR (Chen et al. 2021). The maintenance of acropetal LR positioning, whereby younger roots emerge closer to the root tip, is achieved by restricting LR initiation to a developmental window and repressing ectopic initiation. Recent modeling and in vivo studies suggest that these patterns arise from auxin reflux loops and growth dynamics, with transient auxin peaks in the EZ stabilized by ARF signaling and chromatin modifications (van den Berg et al. 2021; Teixeira et al. 2022).

The *GOLVEN 6/10* (*GLV6/10*) peptide signaling pathway provides an additional layer of regulation: auxin‐induced GLV expression creates a local inhibitory field through PIN7‐mediated auxin export, thereby preventing nearby LR initiation and ensuring proper spacing (Jourquin et al. 2023). Other auxin‐dependent peptide signaling pathways have also been highlighted. For instance, the CEP5 peptide and its receptor XIP1/CEPR1 form a key ligand‐receptor pair involved in the cellular communication required to initiate LR development (Roberts et al. 2016). Additionally, the receptor‐like kinase ACR4 restricts excessive formative cell divisions in both the developing lateral root and the main root tip, thereby ensuring controlled growth (De Smet et al. 2008).

## 3 Auxin‐Modulated Cytoskeletal Reorganization: An Emergence Exit

The emergence of LRs through overlying cell layers requires extensive cellular remodeling of these cells, which is driven in part by auxin‐mediated cytoskeletal reorganization (Stöckle et al. 2022). The nuclear auxin signaling pathway plays a central role in orchestrating these changes by inducing adjustments to cell volume, shape deformations, cytoskeletal rearrangements, and modifications to cell fate and cell wall properties throughout various stages of LR development (Winter et al. 2023).

Nuclear auxin signaling mediated cytoskeleton reorganization is essential for triggering asymmetric cell divisions in the LR founder cells. Disruption of microtubule organization in XPP cells leads to abnormal swelling and interferes with the precise formative divisions required for initiation (Vilches Barro et al. 2019). Cortical microtubules (CMTs) determine the division plane and guide new cell wall deposition, while the actin cytoskeleton facilitates nuclear positioning and membrane trafficking (Winter et al. 2023). Microtubule‐associated proteins (MAPs) further fine‐tune these processes. In Arabidopsis, MAP70‐1 and MAP70‐5 regulate the spatial patterning of cell wall deposition in metaxylem vessels by directing CMT organization and shaping cell wall arches (Pesquet et al. 2011; Sasaki et al. 2023). MAP70‐5, in particular, has emerged as a key player in LR development. While its role as a microtubule bundler is yet to be fully characterized, MAP70‐5 was shown to influence MT polymerization in vitro and is essential for organizing CMT arrays in vivo. Nuclear auxin signaling regulates MAP70‐5 expression in the endodermis during LR initiation, promoting the reorientation of endodermal CMTs from anisotropic to isotropic arrangements around LR founder cells. Interestingly, MAP70‐5 appears to specifically remodel the CMTs facing the XPP in overlying endodermis cells, whereas the CMTs facing the cortex remain isotropic before and after initiation. This reorganization is thought to reduce mechanical resistance to accommodate the expansion growth in the LR founder cells and facilitate formative divisions through the integration of mechanical conflicts between the LR and overlying endodermal cells (Stöckle et al. 2022). The importance of cytoskeletal organization in determining organ morphology is further supported by studies in *Solanum lycopersicum* (tomato), in which the overexpression of *SlMAP70* and *SlIQD21*a results in elongated fruits, whereas loss‐of‐function mutants exhibit a flattened fruit shape. Both proteins were proposed to stabilize CMTs, suggesting that CMT dynamics directly influence tissue shaping in tomato (Bao et al. 2023). Although CELLULOSE SYNTHASE A (CESA) proteins, shown to be crucial for cellulose synthesis in plants (Schneider et al. 2022), have not been directly implicated in spatial accommodation, they are guided by the CMT network, suggesting a potential link between cytoskeletal orientation and cell wall biosynthesis (Winter et al. 2023).

## 4 Breaking Through: Auxin‐Driven Cell Wall Remodeling to Accommodate Expansion Growth

LR initiation requires radial expansion of founder cells, which is facilitated by local cell wall modifications. Auxin‐mediated expression of *EXPANSIN A1* (*EXPA1*) was shown to modify the composition of the pericycle cell walls, thereby allowing for proper radial cell expansion during LR initiation (Ramakrishna et al. 2019). Transcriptomic analyses of auxin‐driven oscillation zones marking LR founder sites revealed the upregulation of genes that encode pectin‐modifying enzymes, such as pectin methylesterases and polygalacturonases (PGs) (Wachsman et al. 2020). This highlights an early and crucial role for pectin remodeling, which is similar to the processes observed at the shoot apical meristem (Peaucelle et al. 2011; Braybrook and Peaucelle 2013). Central to auxin's regulation of root organogenesis are specific transcriptional modules. For LR development, the key module involves ARF7/19 and the LATERAL ORGAN BOUNDARIES‐DOMAIN (LBDs) genes they activate, whereas other root types are initiated by different cooperative ARF‐WOX pairs (Liu et al. 2014; Zhang et al. 2023). These modules directly activate numerous cell wall‐related genes in the overlying cell layers to facilitate LR emergence, including expansins (e.g., *EXPA14, EXPA17*, and *EXPB2*), pectate lyases (*PLA2*), and a subtilisin‐like protease (SBT) (Neuteboom et al. 1999; Swarup et al. 2008; Lee and Kim 2013; Lee et al. 2013; Porco et al. 2016). Receptor‐like kinases (RLKs), such as *MUSTACHES* (*MUS*) and *MUSTACHES‐LIKE* (*MUL*), which are induced by auxin in an ARF7/19‐dependent manner, also promote the expression of *PG, EXPA1*, *EXPA17*, and *XYLOGLUCAN ENDOTRANSGLUCOSYLASE/HYDROLASE PROTEIN 23* (*XTH23*). This is crucial for the transition of the LR from stage I to stage II (Xun et al. 2020). Similarly, auxin upregulates *PG3* in white lupin, several endo‐β‐1,4‐glucanases in poplar, and both *CELLULASE 3* (*CEL3*) and *LEUCINE‐RICH EXTENSIN 2* (*LRX2*) in Arabidopsis (Lewis et al. 2013; Jobert et al. 2021).

Concurrently, the overlying endodermis, characterized by its rigid lignified Casparian strip (CS) and suberin lamellae (SL), undergoes auxin‐mediated remodeling. This includes cell volume loss, localised CS degradation, SL removal, and cutin biosynthesis mediated by Aux/IAA signaling (Vermeer et al. 2014; Ursache et al. 2021; Winter et al. 2023). Auxin regulates suberin dynamics by suppressing genes involved in suberin biosynthesis (e.g., *GDSL‐type esterase/lipase protein 22* (*GELP22*), *GELP38*, *GELP49, GELP51*, and *GELP96*) and promoting the expression of suberin degradation enzymes (e.g., *GELP12*, *GELP55*, and *GELP72*) in the endodermis that overlies the LR (Ursache et al. 2021). This precise modulation of endodermal properties is vital for accommodating LR growth (Vermeer et al. 2014; Ramos et al. 2024). Interestingly, the ablation of an endodermal cell triggers a distinct wound‐healing pathway characterized by restorative cell divisions, rather than facilitating organ formation (Marhavý et al. 2016; Marhava et al. 2019; Hoermayer et al. 2020).

Auxin‐inducible peptide signaling pathways further mediate cell wall remodeling. The *INFLORESCENCE DEFICIENT IN ABSCISSION* (*IDA*)/*IDA‐like* (*IDL*) pathway acts through HEASA/HEASA‐LIKE (HAE/HSLs) receptors to trigger cell wall loosening and separation via a MITOGEN‐ACTIVATED PROTEIN KINASE KINASE 4/5—MITOGEN‐ACTIVATED PROTEIN KINASE 3/6 (MKK4/5–MPK3/6) cascade. In turn, this induces the expression of *EXPA17*, *XTH23*, and *PG* in the overlying tissues (Swarup et al. 2008; Kumpf et al. 2013; Vermeer et al. 2014; Zhu et al. 2019). Conversely, CASPARIAN STRIP INTEGRITY FACTOR (CIF) peptides can inhibit LR progression (Ghorbani et al. 2015; Doblas et al. 2017; Nakayama et al. 2017).

The auxin influx carrier LAX3 amplifies auxin accumulation in the cortex, thereby driving the expression of cell wall remodeling enzymes (Swarup et al. 2008; Péret et al. 2012; Porco et al. 2016). In addition to transcriptional control, auxin also influences cell wall properties through protein phosphorylation and apoplastic pH regulation. Auxin‐activated kinases, including TRANSMEMBRANE KINASEs (TMKs), MPKs, and VIK, link auxin signaling to cell wall modification during LR development (Huang et al. 2019; Fernandez et al. 2020; Kim et al. 2022). For instance, during LR cap cuticle formation, MPK14 and VH1‐INTERACTING KINASE (VIK) phosphorylate and destabilize ETHYLENE‐RESPONSIVE ELEMENT BINDING FACTOR 13 (ERF13), which is a repressor of very‐long‐chain fatty acid (VLCFA) biosynthesis. ERF13 is then targeted for degradation by subsequent ubiquitination by E3 ligases MAC3A/B, promoting cuticle assembly (Lv et al. 2021; Shang et al. 2024; Yu et al. 2024). The ethylene‐responsive transcription factor PUCHI, which is induced by LBD16, positively regulates VLCFA biosynthesis, thereby reinforcing cuticle integrity (Goh et al. 2019; Trinh et al. 2019).

Moreover, auxin promotes apoplastic acidification by activating plasma membrane H^+^‐ATPases (AHAs), a critical process for the activity of cell wall remodeling enzymes such as EXPs, PGs, and XTHs (McQueen‐Mason et al. 1992; Hocq et al. 2017). This activation is often mediated by the phosphorylation of the proton pump and by the action of SMALL AUXIN UP RNA (SAUR) proteins, which stabilize the active AHA complex. Auxin increases AHA levels at the plasma membrane by upregulating transcription, increasing exocytosis, and reducing internalization (Hager et al. 1991; Frías et al. 1996; Paciorek et al. 2005). While TMKs directly phosphorylate and activate AHAs in primary roots (Lin et al. 2021; Yu et al. 2023), it is unclear whether this mechanism functions identically during LR emergence. This balance is counter‐regulated by *RAPID ALKALINIZATION FACTORs* (*RALFs*) and their receptors. For example, *RALF34* and *THESEUS1 (THE1)* restrict pericycle cell division during LR initiation by maintaining cell wall integrity (Haruta et al. 2014; Murphy et al. 2016; Gonneau et al. 2018). Notably, auxin can regulate *RALF* expression, suggesting a finely tuned control of apoplastic pH. Finally, a feedback loop exists whereby cell wall modifications modulate auxin signaling: mechanical cues and pectinase‐mediated cell wall modification upregulate *ARF5* transcription via ERF114 and ERF115 during LR development (Canher et al. 2022). The orchestrated activity of cell wall‐loosening enzymes, pectin distribution patterns (Wachsman et al. 2020), callose deposition affecting intercellular communication (Benitez‐Alfonso et al. 2013; Maule et al. 2013; Sager et al. 2020), and xyloglucan modifications (Roycewicz and Malamy 2014) together enable lateral roots to penetrate parental tissues in an auxin‐coordinated symphony.

## 5 Beyond Arabidopsis: Auxin‐Mediated Spatial Accommodation Across Plant Species

**FIGURE 1:**
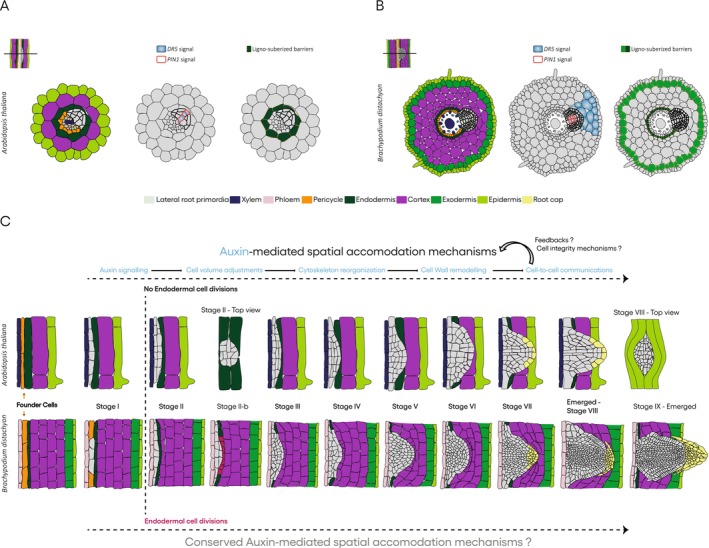
Comparative schematic model of lateral root development in *Arabidopsis thaliana* and *Brachypodium distachyon* , illustrating the interplay between auxin signaling, physical barriers, and cellular dynamics. (A, B) Transverse sections of the two root systems that show auxin activity from observed reporters like DR5 (blue signal) and PIN1 (red line signal), and highlight the ligno‐suberized barriers (yellow/red lines) surrounding the endodermis and exodermis that require active remodeling. (C) A schematic longitudinal view of the sequential stages of LR formation. The developmental progression, which encompasses eight stages in *Arabidopsis* and nine in *Brachypodium* up to full emergence, is driven by auxin‐mediated spatial accommodation mechanisms (light blue). Two distinct cellular strategies are depicted: Top row: The *Arabidopsis* model, which features no endodermal cell divisions. Bottom row: The *Brachypodium* model, characterized by active endodermal cell divisions.

While studies using Arabidopsis have been instrumental in uncovering the cellular and molecular foundations of RSA, anatomical studies across diverse plant taxa reveal considerable variation in LR organogenesis (Xiao et al. 2019). This suggests that, although auxin‐guided spatial accommodation has been well characterized in Arabidopsis, comprehensive and dynamic models remain underdeveloped for many other species, particularly monocots, which have distinct root anatomy (Chen et al. 2018; Motte and Beeckman 2019). Understanding this diversity is essential for elucidating how spatial accommodation mechanisms have evolved and function across the plant kingdom. The evolution of auxin signaling and its downstream effectors has played a key role in diversifying root branching strategies in land plants (Motte and Beeckman 2019; Garg et al. 2022; Omary et al. 2022; Singh et al. 2023; Beeckman and Eshel 2024). Although the core signaling components are conserved, there is divergence in the cellular mechanisms that mediate spatial accommodation during LR emergence, particularly with regard to the roles of the endodermis and exodermis. These layers regulate nutrient transport and environmental responses by depositing hydrophobic biopolymers such as lignin and suberin, forming the CS and SL, respectively (Enstone et al. 2002; Artur and Kajala 2021). In many angiosperms, the formation of LRs involves contributions from the endodermis and pericycle, and in some species, the cortex is also involved. To facilitate LR emergence, some plants reduce endodermal rigidity by promoting cell division and CS loss. For instance, in *Brachypodium distachyon* (Brachypodium), the endodermis appears to undergo dedifferentiation and re‐enter the cell cycle shortly after pericycle division. Furthermore, divisions in the innermost cortical layers may also aid LR emergence. Contrary to Arabidopsis, the auxin‐responsive DR5 reporter in Brachypodium is induced in the endodermis prior to cell divisions and later in the cortex overlying the LR, but was not detected in the phloem pole pericycle prior to asymmetric divisions (De Jesus Vieira Teixeira et al. 2024) (Figure 1A,B). Furthermore, while CSs remain intact in late‐stage LRs, newly formed endodermal cells adjacent to the primordium lack CS domains. This suggests a mechanism for localized wall loosening and tissue accommodation. This contrasts sharply with Arabidopsis, where endodermal cells do not divide during LR emergence. In soybean, the *IDL* genes *GmIDL2a* and *GmIDL4a* are auxin‐inducible genes expressed in developing LR tissues. The two root‐specific *IDL* genes facilitate LR emergence by transcriptionally upregulating a suite of downstream cell wall remodeling genes, thereby loosening the overlying tissues for the developing root primordium to penetrate (Liu et al. 2018).

The exodermis is not universally present in all plant species. Its absence in species such as Arabidopsis and *Hordeum vulgare* complicates efforts to define its evolutionary origins and developmental diversity. For instance, the formation of the exodermis in cotton does not occur in primary roots under standard conditions but is induced by exposure to NaCl (Reinhardt and Rost 1995). In tomato, exodermal differentiation follows a two‐step process involving the initial formation of a polar lignin cap on the inner face of exodermal cells, followed by suberin lamellae deposition to establish a robust apoplastic barrier. This composite barrier is regulated by a rewired genetic program, with the transcription factors *SlSCZ* (*SCHIZORIZA*) and *SlEXO1* (*EXODERMIS1*) controlling polar lignin cap localization, and *SlMYB92* (*MYB DOMAIN PROTEIN 92*), along with *SlASFT* (*ALIPHATIC SUBERIN FERULOYL TRANSFERASE*), being essential for suberin biosynthesis. Mutations of these genes compromise suberin accumulation and affect the plant's response to drought, highlighting the functional relevance of these pathways (Cantó‐Pastor et al. 2024; Manzano et al. 2024). The observation that exodermis cells overlying LRs in Brachypodium display enhanced DR5 reporter activity suggests that, also here, auxin signaling might be required to modify the cellular properties of this resistant cell layer (De Jesus Vieira Teixeira et al. 2024).

There are also significant differences in the spatial origin of LRs. In dicots like Arabidopsis, they originate from xylem pole pericycle cells, whereas in monocots such as *Oryza sativa* , *Zea mays* , and Brachypodium, they initiate from phloem poles (Torres‐Martínez et al. 2022). This anatomical divergence creates distinct challenges for LR emergence, particularly given the hydrophobic barriers formed by the endodermis and exodermis. Dynamic remodeling of the primary cell wall components, cellulose, hemicellulose, and pectin, also plays a critical role. For instance, cross‐sectional autofluorescence microscopy in rice reveals reduced signal intensity of phenolic‐rich compounds, likely due to localized lignin and ferulic acid depletion to accommodate LR growth. Immunolabeling of demethylesterified homogalacturonan further marks sites of wall loosening (Bartley 2016). Notably, some early‐diverging land plants, such as *Selaginella moellendorffii*, exhibit auxin‐independent root branching (Fang et al. 2019). It would be interesting to investigate if root branching in this species would still use similar mechanisms, such as CMT remodeling and which (hormone) pathway would regulate this. Recent studies also suggest that root‐associated microbiota can regulate branching independently of auxin, via ethylene signaling in some cases (Fang et al. 2019; Gonin et al. 2023). These findings point to alternative developmental strategies that may predate or operate in parallel with auxin‐driven pathways.

## 6 Future Perspectives: Charting the Course for Understanding Auxin‐Mediated Spatial Accommodation

Spatial accommodation during root organogenesis fundamentally depends on precise inter‐tissue communication and the orchestrated action of phytohormones, particularly auxin. Although many studies have used the DR5 reporter as a proxy for auxin‐mediated signaling, a recent study has shown that this reporter is likely only reporting a subset of auxin responses. Using protein:DNA binding assays, it was shown that different classes of ARFs have different preferences for binding auxin‐response motives like inverted, everted, or direct repeats of these, with varying spacing between repeats. By combining this with single‐cell analysis of auxin‐mediated gene expression in roots, the authors could show that there is an auxin‐dependent transcriptional code (Martin‐Arevalillo et al. 2025). This will open new avenues for a better understanding of which modules are involved in distinct developmental processes. In addition, it would be important to test whether ARFs of different plant species operate in a similar manner to what was described for Arabidopsis.

For LRs to emerge successfully, the surrounding cell walls must be dynamically remodeled to facilitate tissue expansion and penetration. Cell walls are complex and adaptable networks that undergo significant structural and compositional changes to accommodate growth, rather than being static. Emerging technologies, such as high‐resolution cell wall immunostaining atlases (using multiple plant species) combined with live‐cell imaging, single‐cell transcriptomics, and computational modelling, promise to transform our ability to visualize and understand these dynamic remodeling processes in real time and at cellular resolution. These integrative approaches will enable researchers to analyze how auxin gradients and signaling cascades regulate the specific cell wall modifications necessary for spatial accommodation. Comparative genomics and evolutionary analyses will also be essential for tracing the diversification of key regulators involved in auxin signaling and cell wall remodeling across plant lineages, revealing conserved modules and lineage‐specific adaptations (Figure 1C). Such studies will provide insights into how spatial accommodation mechanisms evolved in response to differing root anatomies and environmental pressures. A critical frontier is to unravel the mechanosensory pathways and cell wall integrity sensing mechanisms that coordinate cellular responses to the mechanical stresses imposed by emerging LRs. Understanding how cells perceive and convert physical forces into molecular signals is essential for deciphering the feedback loops that govern tissue remodeling. Ultimately, deepening our knowledge of these processes will advance fundamental plant developmental biology and facilitate the development of innovative strategies to engineer root systems with improved architecture and resilience. These advancements will be essential for optimizing plant performance in increasingly challenging environments, such as compacted, drought‐stricken, and nutrient‐limited soils.

## Author Contributions

K.B. and J.E.M.V. wrote the first draft of the manuscript. K.B. and C.D.J.V.T. designed the figure. K.B. and J.E.M.V. revised and finalized the manuscript.

## Conflicts of Interest

The authors declare no conflicts of interest.

## Data Availability

Data sharing is not applicable to this article as no new data were created or analyzed in this study.
